# Network Topology of Biological Aging and Geroscience-Guided Approaches to COVID-19

**DOI:** 10.3389/fragi.2021.695218

**Published:** 2021-07-23

**Authors:** Alan Landay, Jenna M. Bartley, Dishary Banerjee, Geneva Hargis, Laura Haynes, Ali Keshavarzian, Chia-Ling Kuo, Oh Sung Kwon, Sheng Li, Shuzhao Li, Julia Oh, Ibrahim Tarik Ozbolat, Duygu Ucar, Ming Xu, Xudong Yao, Derya Unutmaz, George A. Kuchel

**Affiliations:** ^1^ Department of Medicine, Rush School of Medicine, Chicago, IL, United States; ^2^ UConn Center on Aging, University of Connecticut School of Medicine, Farmington, CT, United States; ^3^ Department of Immunology, University of Connecticut School of Medicine, Farmington, CT, United States; ^4^ Engineering Science and Mechanics Department, The Huck Institutes of the Life Sciences, Penn State University, University Park, PA, United States; ^5^ Division of Digestive Diseases, Departments of Medicine, Pharmacology, Molecular Biophysics and Physiology, Rush University Medical Center, Chicago, IL, United States; ^6^ Connecticut Convergence Institute for Translation in Regenerative Engineering, Storrs, CT, United States; ^7^ Department of Kinesiology, University of Connecticut, Storrs, CT, United States; ^8^ Jackson Laboratory for Genomic Medicine, Farmington, CT, United States; ^9^ Department of Genetics and Genome Sciences, University of Connecticut School of Medicine, Farmington, CT, United States; ^10^ Biomedical Engineering Department, Neurosurgery Department, Materials Research Institute, Penn State University, University Park, PA, United States; ^11^ Department of Chemistry, University of Connecticut, Storrs, CT, United States

**Keywords:** COVID-19, aging, systems biology, geroscience, immune aging

## Abstract

Aging has emerged as the greatest and most prevalent risk factor for the development of severe COVID-19 infection and death following exposure to the SARS-CoV-2 virus. The presence of multiple coexisting chronic diseases and conditions of aging further enhances this risk. Biological aging not only enhances the risk of chronic diseases, but the presence of such conditions further accelerates varied biological processes or “hallmarks” implicated in aging. Given the growing evidence that it is possible to slow the rate of many biological aging processes using pharmacological compounds has led to the proposal that such geroscience-guided interventions may help enhance immune resilience and improve outcomes in the face of SARS-CoV-2 infection. Our review of the literature indicates that most, if not all, hallmarks of aging may contribute to the enhanced COVID-19 vulnerability seen in frail older adults. Moreover, varied biological mechanisms implicated in aging do not function in isolation from each other and exhibit intricate effects on each other. With all of these considerations in mind, we highlight limitations of current strategies mostly focused on individual single mechanisms and propose an approach that is far more multidisciplinary and systems-based emphasizing network topology of biological aging and geroscience-guided approaches to COVID-19.

## Introduction

The severe acute respiratory coronavirus (SARS-CoV-2) infects people of all ages; however, severe symptoms, hospitalizations, major complications, and deaths disproportionately afflict older adults (Statistics, 2020; Chen et al., 2021). Nearly 80% of US deaths attributed to COVID-19 have occurred in individuals 65 years and older, with severity and complications increasing with age (Statistics). For example, in China overall best estimates of the case fatality ratio were 20- and 43-times higher in those 60 and 80 years and older when compared to individuals under the age of 60 (Verity et al., 2020). Moreover, according to the most recent CDC figures, aging is a major risk factor with risk of COVID-19 death in individuals 65–74, 75–84, and 85 plus, with risk 90-, 200-, and 630-fold higher, respectively, as compared with 18–29 year olds (Prevention, 2020).

Although chronological age is strongly correlated with COVID-19 severity, biological age—which is a measure of physiological health that is influenced by both chronological age and biological aging processes that promote emergence of multiple chronic conditions—may be a better predictor of COVID-19 severity since comorbidities including hypertension, obesity, diabetes, chronic kidney disease, and heart failure, plus multifactorial geriatric syndromes such as delirium, falls, incontinence, and others represent additional risk factors for frailty with risk of hospitalization and death (Richardson et al., 2020). Even more important than the presence of any one of these individual chronic diseases is the coexistence of multiple comorbidities in the same individual. For example, the accumulation of such deficits, as captured by the Frailty Index, is associated with a greatly enhanced vulnerability in terms of future disability, hospitalization, adverse outcomes, and death (Clegg et al., 2013). Further illustrating the importance of such comorbidities is the finding that among African-Americans, who have been far more vulnerable to being hospitalized or dying from COVID-19 compared to their white counterparts, these striking outcome disparities disappear when adjustments are made for sociodemographic factors and chronic diseases on admission (Price-Haywood et al., 2020).

**FIGURE 1:**
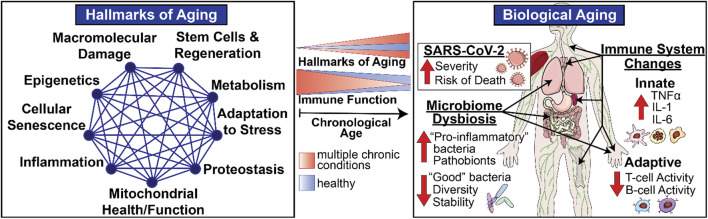
Biological Aging and Immune Function in Late Life. Biological hallmarks or pillars of aging represent multidimensional modifiers and drivers of physiological aging involving varied systems and organs including the immune system. The intensity of these biological drivers of aging increases with chronological aging (blue) resulting in age-related declines in immune responses seen in healthy older adults. However, the scope and magnitude of these varied biological drivers increases in the presence of individual chronic diseases and even more so in frail individuals with multiple chronic conditions (orange), thus providing opportunities for geroscience-guided therapies designed to delay the onset and progression of physiological declines and chronic diseases of aging by target biological pathways.

**FIGURE 2:**
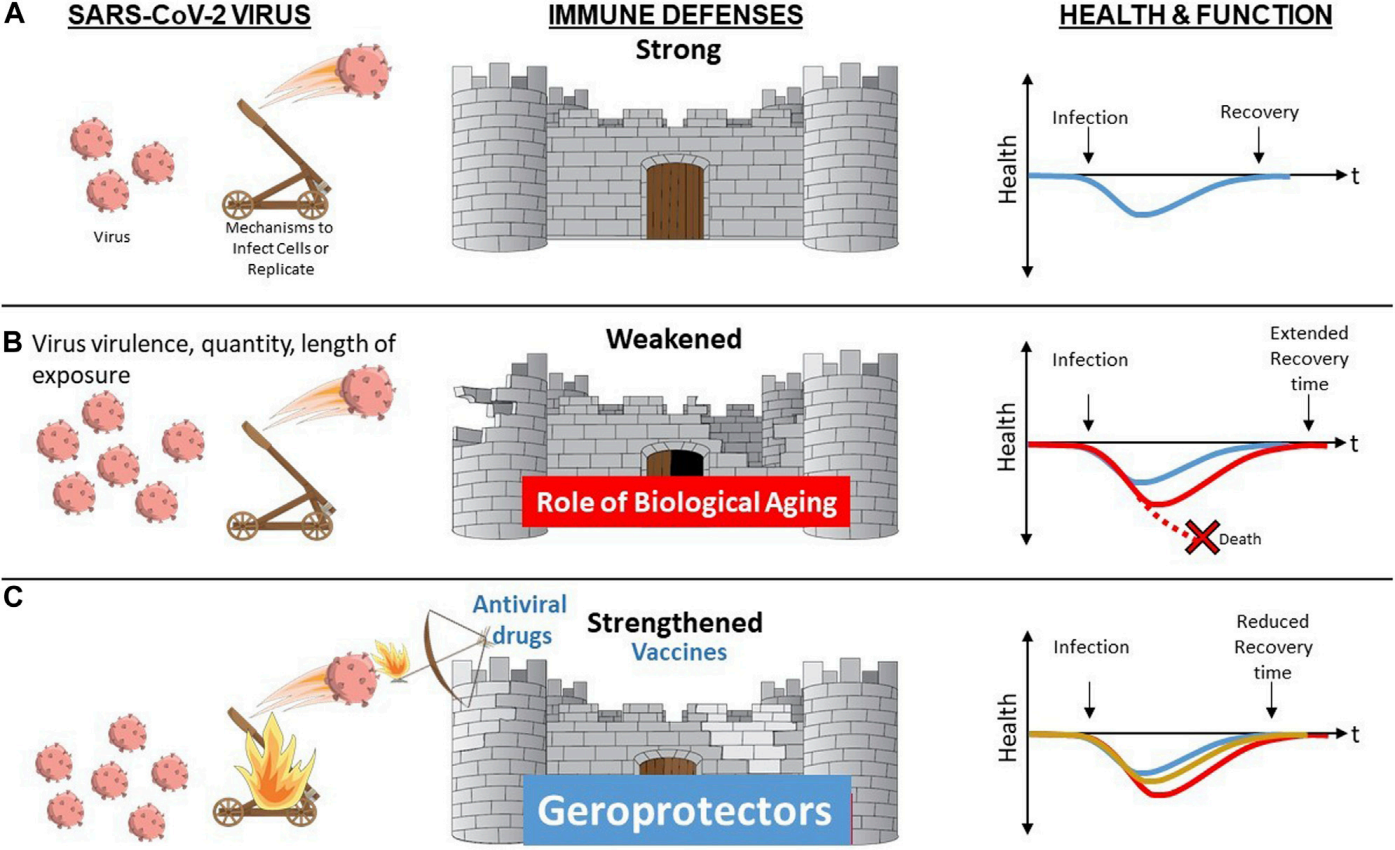
Biological Aging as a Modifiable Risk Factor for Declines in Immune Resilience Upon Challenge with Novel or Altered Pathogen. **(A)** When a resilient immune system is confronted by a novel pathogen such as SARS-CoV-2 virus, it is able to mount a robust primary immune response with rapid and complete clearing of the virus and associated infection resulting in function remaining unaltered or rapidly returning back to baseline (blue). **(B)** Advanced age, especially when accompanied by presence of multiple chronic conditions and/or frailty augment biological aging mechanisms contributing to diminished immune defenses. In the face of increased stressor (greater virus virulence, quantity or length of exposure) and/or diminished immune defenses, resilience mechanisms may be overwhelmed resulting in death (red stippled line) or in a delayed (red) or incomplete return to baseline health and function. **(C)** Vaccine and antiviral drugs offer protection that is typically pathogen-specific. In contrast, geroprotectors that provide the opportunity to boost immune responses by overcoming aging-related declines *via* geroscience-guided therapies may offer benefits irrespective of the type of pathogen involved.

In addition to helping to define the nature of the risk factors that render individuals so vulnerable in the face of COVID-19, these considerations also illustrate the remarkable potential for geroscience-guided therapeutics to alter the underlying natural history and outcomes. The “Geroscience Hypothesis” posits that since aging is the most largest shared risk factor for a myriad of different common chronic conditions, targeting the causes of aging will prevent or delay the onset and progression of multiple chronic diseases, thus expanding years of healthy aging. This potentially transformational approach was enabled by the discovery of distinct “biological hallmarks of aging” that reflect the types of biological processes of aging (Figure 1) that contribute to declines in immune function with aging (Figure 1) and diminished resilience (Figure 2). Moreover, these biological hallmarks of aging do not function in isolation from each other. Instead, they influence each other in a myriad of different ways resulting in what we describe as an interconnected network topology of the biological hallmarks of aging that underlies the Geroscience Hypothesis (Kennedy et al., 2014; Justice et al., 2020).

The impact of aging on diverse aspects of innate and adaptive immune mechanisms is now well recognized (Nikolich-Zugich, 2018), as is the role of these functional changes in increased vulnerability especially when confronted with novel or altered pathogens such as SARS-CoV-2 (Nikolich-Zugich, 2018; Marquez et al., 2020b). These functional declines are most striking in the most vulnerable since the same biological pathways implicated in usual aging represent important shared mediators or drivers of common chronic diseases of aging (Kennedy et al., 2014). Therefore, the presence of multiple chronic conditions in the same individual augments the scope, magnitude, and rate of the impact exerted by biological aging on host defenses and immune function (Figure 1). However, the most striking and clinically relevant changes do not involve static baseline alterations. The most notable impact relates to immune resilience, the ability of the immune system to maintain normal homeostasis in the face of external stressors such as novel pathogens, permitting maintenance of normal function or a rapid and complete return to baseline when challenged (Figure 2) (Hadley et al., 2017; Nikolich-Zugich, 2018).

With the above considerations in mind, individuals who are chronologically older and also experience a large number of varied coexisting chronic diseases and geriatric conditions or syndromes should be viewed as experiencing a form of accelerated biological aging. Although these individuals have greatly enhanced risk of COVID-19, they are the most likely to benefit from geroscience-guided therapies because of the major role played by upregulated drivers of aging. Moreover, an additional benefit of targeting these host-specific factors as opposed to exclusively focusing on SARS-CoV-2 is that this approach is likely to offer benefit while an effective vaccine is being developed, when confronted with a future mutated or entirely novel pathogen, or if a vaccine that works in younger populations is less effective in more vulnerable frail older adults, as has occurred with influenza (Figure 2).

The above considerations highlight the vital importance of systematically addressing how biological aging contributes to declines in host defense mechanisms and immune resilience. To that end, a major focus of this review is the thesis that given the complex interplay between the biology and function of metabolism, microbiome, and the immune system in the context of development, aging, and chronic diseases, it is essential to study biological aging in humans and in a manner that permits an understanding of its impact on relevant metabolic, microbiotic, and immune mechanisms *via* an integrative network topology approach that integrates diverse disciplines and experimental perspectives (Figure 1). Historically, biological research emphasizes and values highly reductionist approaches. Given the complex interactions between these mechanisms, in this review, we propose to enhance this traditional, time-tested approach by adding a systems-based perspective that incorporates a network topology of biological aging and integrates these biological perspectives into a geroscience-guided translational approach to aging (Figure 1).

## Hallmarks of Aging and COVID-19

### Immunosenescence, Inflammaging, and Immunology of COVID-19

As we age, we undergo age-associated remodeling of the immune system toward immunosenescence—a decline in adaptive and innate immunity—and inflammaging—a pro-inflammatory phenotype defined by high circulating levels of pro-inflammatory molecules, such as TNFα, IL-1, and IL-6 (Figure 1) (Franceschi et al., 2007; Bektas et al., 2020; Chen et al., 2021). These overarching changes to the immune system that occur naturally in aging enhance vulnerability of older adults to the SARS-CoV-2 virus and can lead to severe COVID-19 or death. For example, one key factor contributing to COVID-19 severity is the reduced number of naïve T cells in older adults, which diminishes the development of a robust adaptive cellular response to this novel virus (Nikolich-Zugich, 2012). Patients with severe COVID-19 also demonstrate an altered innate immune function, including an impaired interferon type one (IFN-I) response characterized by reduced gene expression and activity (Xia et al., 2020). In a recent study, about 14% of COVID-19 patients who developed serious illness either did not make or mount IFN-1 response to the virus due to genetic defects or development of autoantibodies to IFN-I, whereas only 0.3% of the healthy controls had similar deficiency (Bastard et al., 2020; Zhang et al., 2020b). The reduced IFN-1 activity and response is indeed directly associated with a high blood viral load and a lower SARS-CoV-2 viral clearance (Acharya et al., 2020).

In addition to these overarching immune changes, a number of tissue-specific alterations with aging also need to be considered. In the lung tissue, SARS-CoV-2 can induce a condition known as a cytokine release syndrome—a direct result of a hyperactive innate immune system that cannot be resolved in individuals with a compromised aging immune system. Failure to control SARS-CoV-2 infection results in a sustained cytokine storm with high levels of TNFα, IL-1, and IL-6 in the lungs of individuals with severe COVID-19 (Acharya et al., 2020). Moreover, inflammaging contributes to greater risk for cytokine storm in older adults (Bektas et al., 2020). Importantly, this cytokine storm directly decreases numbers of CD4^+^, CD8^+^, and regulatory (Treg) T cells that are required to control the virus and reduce inflammation (Fajgenbaum and June, 2020). Changes in the innate and adaptive arms of the immune system, a blunted IFN-1 response to control and clear viral infection, and reduced angiotensin-converting enzyme 2 (ACE2) activity in the lungs contribute to increased release of pro-inflammatory cytokines and chemokines, thus promoting inflammation, vascular permeability, and poor outcomes in aged COVID-19 patients (Fajgenbaum and June, 2020; Rydyznski Moderbacher et al., 2020).

Since older people naturally experience inflammaging and immunosenescence, they are more prone to these severe COVID-19 complications and death. Therefore, factors that influence immunosenescence and inflammaging should be considered when evaluating potential therapeutic targets. For example, older males tend to have more innate cell activity (inflammation) but less adaptive cell activity (T- and B-cell function) compared to age-matched older females, which may explain why older males are especially likely to develop severe COVID-19 or die (Marquez et al., 2020a; Marquez et al., 2020b). Understanding why males are predisposed to immunosenescence and inflammaging may provide insights into reducing COVID-19 severity in older males (Marquez et al., 2020a; Marquez et al., 2020b). Given the intrinsic complexities of biological interactions with aging, multidisciplinary studies and systems-based approaches are necessary to evaluate how immunosenescence and inflammaging are related to biological aging, underlying health conditions, and other factors such as gender. These studies will not only reduce the risk of COVID-19 complications in older adults but also alleviate severity of other age-associated diseases.

Moreover, the Immunity Clock consisting of five immune variables—natural killer activity, phagocytosis and chemotaxis of neutrophils, and chemotaxis and proliferative capacity of lymphocytes has been recently proposed as an estimate of the rate of aging of an individual (Martinez De Toda et al., 2021). It remains to be seen to what extent these and other indices may help to predict risk of COVID-19 and other infections.

### Inflammaging and the Microbiome

Studies suggest that chronic, low grade, sterile inflammation (“inflammaging”) is the linchpin associated with age-associated disorders leading to “unhealthy” aging (Franceschi et al., 2007; Franceschi and Campisi, 2014). Although controlled/limited inflammation in response to injury/pathogens is beneficial, chronic or recurrent inflammation can lead to disease. Indeed, there is overwhelming epidemiological evidence that low-grade, chronic inflammation associated with aging accurately predicts morbidity and mortality in humans (Franceschi et al., 2000; Wikby et al., 2006; Larbi et al., 2008; Varadhan et al., 2014). There are several proposed triggers for inflammaging including accumulation of cellular damage across time (i.e., age) leading to chronic NOD-like receptor family pyrin domain containing 3 (NLRP3) inflammasome activation, senescence of immune cells leading to aberrant inflammatory responses, and disrupted microbiome communities (“dysbiosis”) (Furman et al., 2019). Interestingly, these factors are overlapping insomuch that microbiota dysbiosis may be sufficient to drive both inflammasome activation and cellular senescence, suggesting that the age-associated dysbiotic microbiome may be the keystone factor initiating inflammaging (Figure 1).

### Aging GI Track and Microbiota in COVID-19

The microbiome refers to the complex, multispecies communities of bacteria, viruses, fungi, and protozoa that live on and inside the human body (in this review we will use “microbiota” to refer to only the bacterial component of the microbiome). With essential roles in education of the immune system, metabolism, resistance to pathogens, and many others, the microbiome at different body sites likely plays an important role in influencing the host response to viral infection. In addition, significant shifts in the microbiome at nearly all measured body sites occur as individuals age, with a common feature being age-associated dysbiosis—i.e., changes in the “healthy” characteristics of the underlying microbial community such as diversity, stability, or the relative absence of pro-inflammatory microbes or pathobionts (Dejong et al., 2020). We hypothesize that these age-associated changes in the microbiome are an important factor in inflammaging and the increased infection risk and severity of COVID-19 observed in older adults. Here, we will briefly discuss potential roles of the microbiome of the gut and airway and their potential interactions in aging-related infection risk.

#### The Aging Intestinal Microbiome

The intestinal microbiome plays a critical role in promoting health and disease; therefore, age-associated changes in the microbiome can have dramatic effects on health. The “mature” microbiome is relatively stable in healthy adults until approximately 65 years of age. The microbiota communities in people of advanced age (i.e., >65 years) are characterized by reduced microbial diversity, reduced beneficial commensal bacteria, and an increase in the abundance of pathobionts (Hopkins et al., 2001; Hopkins et al., 2002; Mariat et al., 2009; Makivuokko et al., 2010; Tiihonen et al., 2010; Claesson et al., 2011; Biagi et al., 2012; Claesson et al., 2012; Duncan and Flint, 2013; O'toole and Jeffery, 2015; Rondanelli et al., 2015; Kato et al., 2017; Vaiserman et al., 2017). While the specific bacterial populations impacted by age differ from study to study, overall, these studies confirm that the microbiota of people of advanced age tend to be “pro-inflammatory.” The causative factors that initiate these changes are not clear but may include alterations in the types of food that are consumed, changes in nutrient absorption and GI motility, antibiotic use, or alterations in circadian rhythms and subject mobility that leads to less exercise and activity. Stress associated with loneliness and isolated living can also contribute to age-associated dysbiosis (Dejong et al., 2020). Age-associated intestinal microbiota dysbiosis is associated with frailty in humans (Stehle et al., 2012; Ghosh et al., 2015; O'toole and Jeffery, 2015). Data in rodents provide a compelling argument that the intestinal microbiome is the lynchpin that promotes aging: germ-free (GF) mice live longer than their conventionalized counterparts and fecal microbiota transfer from old mice into GF or young mice disrupts the intestinal barrier, increases circulating cytokines, and promotes immune dysfunction (Fransen et al., 2017; Thevaranjan et al., 2017; Grosicki et al., 2018; Kim and Jazwinski, 2018). Typically, the intestinal barrier restricts the passage of pro-inflammatory luminal contents from reaching the intestinal mucosa and the systemic circulation; however, defects in intestinal barrier function can lead to robust inflammation and the promotion of inflammation-mediated disease (Kim and Jazwinski, 2018).

In addition to age-related changes in immune function likely mediated in part by microbial dysbiosis, emerging evidence shows that the SARS-CoV-2 virus directly infects intestinal epithelial cells and triggers host inflammatory responses (Lamers et al., 2020). Indeed, recent studies have shown viral shedding in the stool of patients with COVID-19 even when nasopharyngeal swabs are negative for the virus, and this shedding can last for 40 days (Zheng et al., 2020c). Other studies show that at least 10–30% of patients with COVID-19 have intestinal symptoms including diarrhea, further supporting SARS-CoV-2 infection of intestinal epithelial cells (Lamers et al., 2020). Thus, it is highly plausible that the intestinal microbiota can modify the interactions between the intestinal epithelial cells and SARS-CoV-2 which may modify the rate and duration of viral infection and its short- and long-term consequences. We posit that a potential mechanism for increased risk of SARS-CoV-2 infectivity and severe COVID-19 in the elderly involves the interaction of “pro-inflammatory” dysbiotic intestinal microbiota. We recently demonstrated that systemic levels of fungal and bacterial products translocated from the gut correlate with COVID-19 severity (Giron et al., 2020). Thus, identification of marker molecules can provide diagnostic and prognostic biomarkers for the disease that are specific to the age group.

#### The Airway Microbiome

In addition to the intestinal microbiome, the microbiome of the upper respiratory tract, beginning with the nasal cavity, nasopharynx, and oropharynx, plays an important role in the full morphogenesis of the respiratory tract. The diversity of microbes inhabiting these sites is largely a characteristic of the microbiota of the skin and oral cavity, and drives the development of tissue-specific immunity and colonization resistance to pathogens. Recent research has also identified specific microbial interactions that change a host’s viral susceptibility. For example, immune priming of human macrophages with lipopolysaccharide reduces the ability of influenza A virus to infect those cells (Short et al., 2014), and commensal microbes are essential to mount CD4 and CD8 T cell and antibody responses in the lung (Herbst et al., 2011). Nasal host–microbe interactions can also affect lung viral susceptibility. Intranasal inoculation of an opportunistic pathogen, *Staphylococcus aureus,* dampened influenza virus-induced inflammatory responses (Wang et al., 2013), while inoculation of a commensal *Lactobacillus* species resulted in protection against pneumovirus infection in the lungs of mice (Rice et al., 2016). Finally, lung commensals are associated with lymphocyte numbers, lung inflammation, and a reduced macrophage response in human bronchoalveolar lavage fluid (Segal et al., 2016). Collectively, there is significant precedent that the airway microbiome can strongly affect local viral infection and subsequent immune response.

However, few studies have been conducted to understand how the microbiome of the upper respiratory system changes during age. Our recent study compared the skin, oral, and gut microbiomes of older adults >65 years of age, including individuals living in the community as well as skilled nursing facilities (Larson et al., 2021). We found that significant changes occur in the nasal and oral microbiota with increasing age, including increased representation of pathobionts such as *S. aureus*, reduced immunomodulatory commensals characteristic of those skin sites such as *Staphylococcus epidermidis* (Linehan et al., 2018), and lower microbial diversity. Future research is needed to determine to what degree these broad microbiome shifts contribute to an altered immune milieu that may increase susceptibility to viral infection or propagation of viral responses.

### Bacteria Influence Viral Infectivity

SARS-CoV-2 is thought to pass through the mucus membranes, especially nasal and larynx mucosa. These surfaces are inhabited by a commensal microbiome that may influence susceptibility to SARS-CoV-2 infection. Direct virus–bacteria interactions are observed between bacteria and viruses such as influenza A virus, picornaviruses (e.g., poliovirus, coxsackieviruses, echovirus 30, mengovirus, Aichivirus), human noroviruses, and mammalian orthoreovirus (reovirus) (Kuss et al., 2011; Robinson et al., 2014; Li et al., 2015; Almand et al., 2017; Berger et al., 2017; Waldman et al., 2017; Erickson et al., 2018; Aguilera et al., 2019; David et al., 2019; Rowe et al., 2019). These interactions appear to be biologically meaningful since bacteria–virus interactions can influence virion stability and infection of eukaryotic cells. Virus–bacteria interactions can protect viruses from the destructive effects of factors such as heat (thermostability) and bleach (Kuss et al., 2011; Li et al., 2015; Almand et al., 2017; Berger et al., 2017; Aguilera et al., 2019). On the other hand, virus–bacteria interactions can also disrupt virus integrity and reduce infectivity (Orchard et al., 2016; Shi et al., 2019). These viral–bacterial interactions also appear to be important for coronaviruses. *Bacillus subtilis* (a Gram-positive bacteria defined as an obligate or facultative anaerobe) disrupts coronavirus integrity which is thought to be due to *Bacillus subtilis* production of surfactin, a cyclic lipopeptide with membrane disruptive properties (Cochrane and Vederas, 2016; Johnson et al., 2019). Thus, it is possible that commensal bacteria on the surface of the nasal and oral cavities influence susceptibility to SARS-CoV-2 infection.

### Epigenetics of Aging and COVID-19

Epigenetic landscape of cells plays critical roles in precisely regulating gene expression programs (Akalin et al., 2012). These include histone modification and DNA methylation marks that determine the accessibility of the DNA and chromatin interactions between regulatory elements that dictates the 3D structure of the genome (Gibcus and Dekker, 2013). Epigenetic marks are established during development and are stably inherited during mitosis. These marks and landscapes can change during aging (Ucar et al., 2017; Marquez et al., 2020a), and with complex diseases, such as cancer (Li et al., 2016a; Li et al., 2016b). However, it is unclear if epigenetics can be used as predictors of COVID-19 severity, how COVID-19 infections change the epigenetic landscapes of cells, and whether therapies reversing epigenetic changes can be used to treat COVID-19 patients.

### Epigenetic Clocks as Predictors of COVID-19 Disease Severity

Although older adults are disproportionately affected by COVID-19, chronological age alone is not sufficient to predict COVID-19 severity (Atkins et al., 2020; Zhang et al., 2020a; Guan et al., 2020; Li et al., 2020; Richardson et al., 2020; Yang et al., 2020). Since biological age outperforms chronological age in predicting lifespan and health-span, the composite biomarker measures that indicate biological age (Jylhävä et al., 2017) may be applied to predicting COVID-19 disease severity. Epigenetic clocks are among the most prominent biological age predictors, developed using thousands of CpG sites for an aging surrogate, e.g., chronological age or mortality (Horvath and Raj, 2018). Interestingly, obesity (Horvath et al., 2014), smoking (Demeo et al., 2016), and being male (Horvath et al., 2016) are factors associated with the accelerated ticking of these clocks, and are also associated with COVID-19 disease severity. Furthermore, genome-wide association studies of two popular epigenetic clocks (Hannum et al., 2013; Horvath, 2013) identified distinct metabolism- and immune-related pathways underlying the accelerated epigenetic age (Lu et al., 2018; Gibson et al., 2019). However, it is to be seen whether existing epigenetic clocks—most of which are driven from blood epigenomic maps—can be effective in predicting COVID-19 disease risk at the individual level.

### Potential Epigenetic Therapies to Combat SARS-CoV-2 Infections

While epigenetics has potential to predict COVID-19 disease severity, it may also provide therapeutic targets for those with the SARS-CoV-2 virus. ACE2 is a receptor for the SARS-CoV-2 spike glycoprotein (Hoffmann et al., 2020) and is abundantly expressed in the epithelia of the lungs and small intestine, which are possible entry routes for the virus (Hamming et al., 2004). Data from 700 patients with comorbidities associated with severe COVID-19 disease (e.g., smoking, COPD, hypertension) showed that ACE2 gene expression is elevated in the lung cells of these patients compared to control individuals (Pinto et al., 2020). Furthermore, ACE2 expression was significantly correlated with the expression of several histone-modifying genes (e.g., HAT1, KDM5B), which can directly affect gene expression levels by modifying histone marks. For example, KMD5B is a demethylase for lysine four of histone H3—a modification found on the active genes’ promoters including that of ACE2 in lung cells (Pinto et al., 2020). Similarly, DNA hypomethylation and increased expression of ACE2 was reported in a subset of CD4^+^ T cells in systemic lupus erythematosus patients (Sawalha et al., 2020). These data suggest that ACE2 expression might be elevated in individuals who are at high risk for COVID-19 due to epigenetic remodeling. Further research is needed to confirm these preliminary findings; however, these can open doors to potential epigenetic therapies, including molecules that can inhibit KDM5B that have been studied in the context of cancer (Rasmussen and Staller, 2014; Pippa et al., 2019).

In alignment with potential epigenetic therapies for COVID-19, affinity purification-mass spectrometry (AP-MS) data uncovered 332 interactions between SARS-CoV-2 proteins and human proteins that might serve as drug targets. The main protease of SARS-CoV-2 (Nsp5) interacted with the epigenetic regulator histone deacetylase (HDAC2) (Gordon et al., 2020), whereas the viral transmembrane protein E interacted with the bromodomain-containing proteins (i.e., BRD2, BRD4) (Gordon et al., 2020). BRD2 binds to the acetylated histones to regulate gene expression. Hence, it is speculated that virus protein E can mimic the histone to disrupt BRD2–histone interactions, thereby inducing changes in the gene expression programs of the host cells. Future studies are needed to establish whether BRD4 inhibitors (e.g., JQ1) or HDAC inhibitors may attenuate the actions of SARS-CoV-2 proteins inside human cells.

### Metabolism, Obesity, and Cardiovascular Disorders in COVID-19

Cardiovascular and metabolic diseases—including hypertension, diabetes, and obesity—are among the top comorbidities of COVID-19 patients (Atkins et al., 2020; Zhang et al., 2020a; Guan et al., 2020; Li et al., 2020; Richardson et al., 2020; Yang et al., 2020). Given that diabetic and obese patients have dysregulated immune baselines (Donath and Shoelson, 2011), their encounter with the SARS-CoV-2 virus bears both metabolic and immunological consequences. An animal study of MERS-CoV indicated that comorbid type 2 diabetes alters the immune response profile after the infection, including an increase in IL-17a levels, raising a possible explanation for why diabetic COVID-19 patients suffer more severe lung pathology (Kulcsar et al., 2019). To further complicate the disease and the treatment, the infection by coronaviruses may also cause acute diabetes. A study from the 2003 SARS pandemic that was caused by another coronavirus (SARS-CoV-1) reported that nondiabetic patients became diabetic during hospitalization (Yang et al., 2010). The authors suggested that this acute diabetes was a result of coronavirus damaging pancreatic islet cells. It is not yet known if SARS-CoV-2 has the same mechanism of pathogenesis, but it is possible. Although the role of hyperglycemia is not yet clear during the pathogenesis and prognosis of SARS-CoV-2, metabolic control is clearly critical for patient management, especially in diabetic patients (Hill et al., 2020; Zhou and Tan, 2020).

While metabolic and cardiovascular chronic conditions may impact COVID-19 progression on their own, metabolism of the drugs used to treat these chronic conditions of aging may also have effects on COVID-19. Hypertension is among the top comorbidities for COVID-19 severity. The ACE2 protein, reported as a receptor to SARS-CoV-2 infection (Zhou et al., 2020), acts as a key regulatory point for the angiotensin system, balancing hormonal modulation of blood pressure (Bornstein et al., 2020). Because multiple pharmacological drugs for hypertension target this pathway and affect either the availability or expression level of ACE2, we are presented with enormous complexity in the susceptibility, pathogenesis, and treatment of COVID-19 (Bosso et al., 2020). While the elderly population is known to have higher mortality rate in COVID-19, those anti-hypertension drugs are also more prevalent in this population. Detailed studies of drug metabolism and immunometabolism are necessary to understand these complex interactions in older patients with comorbidities.

Although the specific mechanisms of metabolic disorders that impact COVID-19 severity are not clear, evidence indicates that metabolic dysregulation and the immune response have strong ties in COVID-19 patients. The immune response in severe cases of COVID-19 commonly showed lymphopenia with low numbers of multiple cell types including CD4^+^ and CD8^+^ T cells (Huang et al., 2020; Qin et al., 2020; Xu et al., 2020). Analyses of blood cells from COVID-19 patients also showed significantly increased levels of exhaustion markers in CD4^+^ and CD8^+^ T cells (Zheng et al., 2020a; Zheng et al., 2020b; Diao et al., 2020). This exhausted state is likely accompanied by systemic metabolic dysregulation. Additionally, early reports indicate that massive changes in the metabolome and lipidome occurred in patient blood (Shen et al., 2020; Wu, 2020). Such mass spectrometry-based, high-throughput metabolic data will accumulate quickly as people carry out patient sample analyses and experimental studies that will provide information on both cellular metabolic programs and systemic chemical signals. For one cellular metabolic program, immunometabolism, a large body of recent works have unveiled how specific metabolic pathways are tied to the development and function of immune cells at different stages, including activation and exhaustion (Buck et al., 2017; Kishton et al., 2017; Lee et al., 2018). Changes in systemic chemical signals include dysregulation of lipid and glucose metabolism by viral infection, changes in amino acids from compromised organ functions, and eicosanoids that are commonly accompanied by cytokine storms. Given the interconnected nature of metabolism and immune function, multidisciplinary, systems-based investigations may illuminate potential therapeutic targets that can alleviate metabolic and immunologic stress in older individuals with COVID-19.

### Role of Mitochondrial Dysfunction and Autophagy

Since mitochondria regulate metabolism, cellular homeostasis, aging, innate and adaptive immunity, apoptosis, and other signaling pathways, investigating mitochondria dysfunction with aging may illuminate targets for alleviating chronic conditions and COVID-19 severity. Aging leads to declines in mitochondrial function which impairs respiratory chain efficacy by increasing electron leakage and decreasing ATP generation, resulting in the accumulation of reactive oxygen species (ROS) and the decrease of detoxifying systems. This could induce severe oxidative damage to mitochondrial components, thereby accelerating the development of age-related chronic diseases including acute and chronic inflammatory diseases (Brookes et al., 2004; Naik and Dixit, 2011; Breda et al., 2019; Breda et al., 2019). Mitochondrial ROS could activate the redox-sensitive factor nuclear factor-κB (NF-κB) pathway and induce NLRP3 inflammasome activation that could work together to activate inflammatory cytokines, thus leading to overstimulation of the inflammatory response (Amma et al., 2005; Cillero-Pastor et al., 2008; Chung et al., 2009; Naik and Dixit, 2011; Breda et al., 2019). Although it is unclear, excessive production of mitochondrial ROS may cause oxidative damage in people who have COVID-19; therefore, a role for mitochondria in mediating enhanced vulnerability as a result of aging and lifestyle factors has been proposed (Nunn et al., 2020).

Autophagy is the self-destructive process which removes unnecessary or dysfunctional cellular components. Autophagy can control inflammation through inhibiting NLRP3 inflammasome activation or the macrophage-mediated clearing system by removing mitochondrial ROS production (mitophagy) (Kepp et al., 2011; Nakahira et al., 2011). Since autophagy declines with aging, decreases and mutations in the expression of autophagy-related genes—specifically PINK2 and Parkin in mitochondria—have been shown in different inflammatory diseases and aging processes (Ding and Yin, 2012; Leidal et al., 2018). Therefore, it is easy to speculate that COVID-19-induced disruption of mitophagy may affect innate immunity and reduce the levels of host machinery that stages the anti-inflammatory and antiviral responses (Garcia-Perez et al., 2020).

### Understanding Complex COVID-19 Pathogenesis in Humans *via* 3D Bioprinting and Organoids

Clearly, the hallmarks of aging—including senescence, inflammation, epigenetics, metabolism, and mitochondrial dysfunction—each have a role in advancing biological age; however, based on our literature review, the effects of these hallmarks are strongly interdependent. The SARS-CoV receptor, ACE 2 (Bosso et al., 2020), is expressed by alveolar epithelial type II cells and ciliated cells in the lungs and in the brush borders of the intestinal enterocytes (Bosso et al., 2020). Not only will it take truly interdisciplinary and systems-based approaches to understand how COVID-19 affects the elderly, but it will also require the development of novel, physiologically relevant model systems.

SARS-CoV-2, which causes acute respiratory symptoms and severe lung injury, is detected in the upper respiratory tract of humans, implying that the nasopharynx is a site of replication. Although SARS-CoV-2 is one of the many zoonotic pathogenic viruses that jumped to humans in the last 2 decades, studies on virus pathogenesis and transmission are limited by a lack of *in vitro/ex-vivo* models to host lung and intestinal tissues.

However, these models do not recapitulate the complex 3D anatomy of the lung matrix parenchyma which requires connections of alveolar units to airways surrounded by endothelium (Suki et al., 2011), further complicated by the dual multivasculature (Grigoryan et al., 2019). Studies on lung-specific diseases have largely been autopsies performed on lungs of patients who died or conducted in animal models. Animal models—particularly, telomerase null, senescence accelerated mice, or klotho null mice (Navarro and Driscoll, 2017)—are used to study the underlying molecular and cellular changes in an aging lung, leading to the progressive loss of regenerative stem cells. However, these do not always recapitulate human physiology (Bracken, 2009). A robust *in vitro* engineered human tissue model will bridge the gap in our current knowledge of aging lung immune responses against airborne viral infections such as COVID-19. 3D bioprinting is a promising technological advancement, which facilitates direct printing of biologically relevant materials and cells (Peng et al., 2016; Datta et al., 2020). It enables the fabrication of complex geometries and defeats the concerns related to cell death, insufficient cell density compared to physiological relevance, and cell attachment in conventional 3D printing procedures. Substantial relevance of bioprinting is particularly note-worthy in lung tissue engineering because of its potential to fabricate hollow structures with multicellular layers. With significant materialization of lung bioprinting, many techniques surfaced—although most of these are in their infancy and far from clinical translation. The future of successful 3D lung bioprinting relies on resolving set of three key challenges: identifying reliable cell sources for the fabrication of the airways, from the ciliated epithelium to alveolar epithelium; maintaining the openness of the lumen at all scales of the lung, especially the vasculature during culture; and providing adequate gas and fluid perfusion, in tandem with physiologically relevant breathing stimulus.

Until recently, many research endeavors exploring mono- or multilayer *in vitro* lung cultures (Braian et al., 2015; Miller and Spence, 2017) are focused on the recreation of epithelial cell culture at the air/liquid interface. The first effort in the development of a breathing lung was done using poly (dimethylsiloxane) (Huh et al., 2010), which was further explored by bioprinting alternating layers of epithelial and endothelial cell-laden Matrigel (Horvath et al., 2015). Lewis et al. focused on the recapitulation of the alveolar epithelial morphology and used poly-ethylene glycol (PEG) microspheres to fabricate hollow, physiologically relevant epithelial cysts, mimicking the scale of the alveolus (Lewis et al., 2015). Improving more on a complex and physiologically relevant geometry, a breathing PEG diacrylate lung model with dual multivasculature and tidal air ventilation was printed using stereolithography (Grigoryan et al., 2019). Despite the low resolution of 300 μm and a lack of functional relevance, this was a significant advancement toward clinical translation of lung tissue.

Thus, it seems apparent that despite respiratory problems dominating the clinical symptoms of COVID-19, gastrointestinal problems have been observed in a smaller group of patients (22%), In humans, the most abundant expression of ACE2 occurs in the brush borders of the intestinal enterocytes (Qi et al., 2020), with a recent study demonstrating clinical evidence of the intestine as a target organ for SARS-CoV-2 (Lamers et al., 2020). The intestine and its microbiota have emerged as important triggers/modifiers for diseases of aging where inflammation and metabolic dysfunction play a central role in their pathogenesis (Heintz and Mair, 2014; Buford, 2017). Indeed, gut-derived inflammation as a consequence of intestinal leak to bacterial pro-inflammatory products, such as endotoxins, is a major contributor of host inflammatory response to pathogens, including respiratory viruses (Bischoff et al., 2014; Buford, 2017). Recent advances in generating intestinal organoids (Bartfeld and Clevers, 2017) provide an exceptional opportunity to investigate the molecular and cellular mechanisms of aging gut and its contribution to disease susceptibility such as COVID-19 in the elderly. Unlike lung, intestinal tissue can easily be obtained from healthy and diseased subjects through endoscopic procedures. Several studies show that mouse and patient-derived intestinal organoids maintain host phenotypes (Schwank et al., 2013; Bartfeld and Clevers, 2017; Forsyth et al., 2017), and thus organoids can be used to interrogate mechanisms and establish causal links using loss/gain of function approaches (Schwank et al., 2013; Drost et al., 2017). Furthermore, intestinal cell monolayers generated from organoids will provide opportunities for coculturing intestinal cells with bacteria, therapeutic agents, viruses (at the luminal side), and immune cells/therapeutic agents in the basolateral side of the monolayer to interrogate these biologically relevant interactions. We believe intestinal organoids, generated from sigmoid biopsy through limited sigmoidoscopy (Sato et al., 2011; Forsyth et al., 2017; Hibiya et al., 2017) in SARS-CoV-2-infected elderly and young patients, will provide excellent opportunities to investigate the potential impact of intestine biology on acute and long-term impact of COVID19 disease. Ultimately, creating robust intestinal and lung models made with aged human cells will not only improve biological understanding of COVID-19, but they will also inform and provide *in vitro* testbeds for treating COVID-19 and future pathogens.

## Vaccine Development for Emerging Pathogens in Older Adults

In the face of this pandemic, we have seen the development, testing, and recent approval of two new vaccines occur with unprecedented speed (Anderson et al., 2020; Polack et al., 2020). A variety of factors, including the use of novel of mRNA-based technology, allowed a vaccine development process that normally takes years to be compressed into mere months. Unlike other similar efforts (Helfand et al., 2020), these trials did include some older adults (Anderson et al., 2020). There is no doubt as to the importance of establishing safety and immunogenicity in relatively healthy older adults (Anderson et al., 2020). At the same time, the vaccines remain unstudied in the most vulnerable population—frail older adults with multiple chronic conditions, especially those who reside in long-term care facilities where nearly half of COVID-19 deaths in the US have occurred (Yi et al., 2020).

The hurdles to developing a novel COVID-19 vaccine for older adults are numerous, especially since proper functioning of the immune system is negatively impacted by aging. Progressive declines in immunity, known as immunosenescence, are observed in both the innate and adaptive arms of the immune system. One of the most prominent changes with increasing age is the decline in the naïve CD4 T-cell population and the corresponding increase in the memory T-cell population (Thome et al., 2014). This is especially important because naïve CD4 T cells are essential for the response to novel emerging pathogens and newly developed vaccines. Because of immunosenescence, older adult immune responses to novel vaccines are often reduced when compared to younger people.

Strategies to enhance vaccine immunogenicity in older adults include increasing antigen dose, using more potent adjuvants, and modifying vaccine composition (Lefebvre and Haynes, 2013). With regards to protein-based and inactivated vaccines, increasing antigen dose aims to overcome deficits in antigen presentation that can arise with aging. Indeed, the high-dose inactivated influenza vaccine significantly increases antibody titers and seroconversion rates in older adults (Diazgranados et al., 2014; Tsang et al., 2014; Gravenstein et al., 2017). Adjuvants are also useful to both boost and differentially skew immune responses to a protein or inactivated virus vaccine. This is especially important since immune responses in older individuals may be dysregulated, skewing toward more pro-inflammatory responses that can exacerbate diseases rather than promote protective responses (Mcelhaney et al., 2016). In fact, a newly developed adjuvanted seasonal influenza vaccine elicits significantly higher antibody production and enhances overall protection in older adults (Van Buynder et al., 2013; Frey et al., 2014). Modifying vaccine composition is another approach to enhance vaccine efficacy. For example, live attenuated influenza vaccines enhance CD8 T-cell responses in older adults, which may be less impacted by aging when compared to antibody responses (Mcelhaney et al., 2006; Mcelhaney et al., 2009; Merani et al., 2018). Since viral-vector COVID-19 vaccines are currently being developed (Callaway, 2020), this bodes well for their efficacy in older adults. It is important to note, however, the target of these vaccines is also crucial in providing protection to older adults.

Recent work has shown that in patients who recovered from COVID-19, not surprisingly, neutralizing antibody titers correlate with anti-Spike (S)-receptor-binding domain (S-RBD) IgG, but not anti-nucleocapsid (NP) protein IgG levels. A spike-specific T-cell response was also found in these patients (Ni et al., 2020) suggesting that a vaccine that targets the spike protein may produce both cell-mediated and humoral immunity, which would be particularly beneficial to older adults. More research, however, has suggested that a vaccine consisting of spike protein alone would elicit a similar CD4 T-cell response to natural immunity, but may not represent natural CD8 T-cell responses. In the total CD8 T-cell responses, the spike protein was not the clear dominant epitope with M protein and other antigens comprising nearly 50% of the response (Grifoni et al., 2020), suggesting inclusion of other class I epitopes would enhance vaccine responses to mimic natural infection in CD8 T cells. Indeed, recovered middle-aged and older adults had greater neutralizing antibody titers compared to young adults (Wu et al., 2020), leading to more questions regarding the relation of age and humoral responses to COVID-19 that need to be considered during vaccine development.

Additionally, novel, more geroscience-based approaches may be particularly promising by targeting pathways implicated in the overall aging process rather than specific deficits in the immune response. Targeting metabolic deficits that develop with aging *via* inhibition of mTOR with a rapamycin analogue resulted in improved flu vaccine antibody responses (Mannick et al., 2014b) and enhanced overall immune function (Mannick et al., 2018) in older adults. Other strategies, such as administration of senolytic drugs that can reverse many age-related diseases (Xu et al., 2018; Yousefzadeh et al., 2018), may also be an option for improving overall immunity and enhancing vaccine effectiveness. Since COVID-19 is more severe in those with pre-existing comorbidities, senolytics may represent a unique way to rejuvenate multiple systems of the body to result in more resilient responses to both infection and vaccination in older adults.

## Role of Senolytics and Other Geroscience-Guided Therapies

The biological process of aging influences nearly all aspects of cellular function with a measurable impact on the physiological performance of varied organ systems, including declining immune resilience with aging that results in increased susceptibility to infections and resulting mortality. Described as hallmarks (Lopez-Otin et al., 2013) or pillars (Kennedy et al., 2014) of aging, biological aging impacts processes as diverse as stem cell exhaustion, altered intercellular communication, genomic instability, telomere attrition, epigenetic alterations, loss of proteostasis, deregulated nutrient sensing, mitochondrial dysfunction, and cellular senescence.

Until the emergence of the Geroscience Hypothesis (Sierra and Kohanski, 2017), a major gap remained between biological and more clinical strategies to study and intervene in the aging process. This revolutionary approach to aging and the improvement of human health throughout the lifespan, which is rapidly moving from the stage of a hypothesis to the development and testing of novel geroscience-guided therapies, is based on a series of scientific observations and discoveries. First, advanced age represents by far the largest risk factor for most adult chronic diseases. Second, this risk factor is further compounded by the presence of multiple coexisting chronic diseases, geriatric syndromes, and conditions. Third, many animal model studies have demonstrated that therapies designed to target one or more biological hallmarks of aging are capable of preventing or slowing the onset of varied chronic diseases. Fourth, mTOR inhibitors can extend lifespan (Harrison et al., 2009) and alleviate age-related diseases (Li et al., 2014) in mice with demonstrated benefits in improving flu vaccine responses (Mannick et al., 2014b) and preventing respiratory infections (Mannick et al., 2018) in older adults. At the same time, senolytics—drugs designed to clear senescent cells—have demonstrated safety and initial feasibility in early proof of concept trials targeting idiopathic pulmonary fibrosis (Justice et al., 2019a) and diabetic kidney disease (Hickson et al., 2019). Finally, extensive evidence has shown that older diabetics taking metformin have lower rates of cancer, dementia, heart disease, and overall mortality when compared to diabetics taking other oral hypoglycemic drugs (Justice et al., 2018b). Metformin, unlike other oral hypoglycemic agents, targets most of the biological hallmarks of aging (Kulkarni et al., 2020). With these considerations in mind, TAME (Targeting Aging with Metformin), a first of its kind clinical trial, will seek to test the hypothesis that an intervention targeting different biological hallmarks of aging will delay the onset and progression of an aggregate of chronic diseases, including cardiovascular disease, cancer, and dementia, with additional impact on relevant functional measures and biomarkers (Justice et al., 2018a; Justice et al., 2018b; Kulkarni et al., 2020).

Cellular senescence is an especially attractive therapeutic target for retarding the aging process and improving immune function. Cellular senescence refers to the essentially irreversible proliferation arrest that occurs when cells experience a range of stresses (Campisi and d'Adda di Fagagna, 2007). With aging, such cells accumulate in multiple tissues (Zhu et al., 2014; Schafer et al., 2016; Xu et al., 2018) including lung (Campisi, 2016). The roles of senescent cells in various age-related pathological conditions have been extensively examined, including physical dysfunction (Xu et al., 2015b; Xu et al., 2018; Wang et al., 2020), osteoporosis (Farr et al., 2017), adipose tissue dysfunction (Xu et al., 2015a), diabetes (Palmer et al., 2019), osteoarthritis (Jeon et al., 2017; Xu et al., 2017), cardiac dysfunction (Baker et al., 2016), kidney dysfunction (Baker et al., 2016), vasomotor dysfunction (Roos et al., 2016), atherosclerosis (Childs et al., 2016), liver steatosis (Ogrodnik et al., 2017), pulmonary fibrosis (Schafer et al., 2017), stem cell dysfunction (Xu et al., 2015a; Chang et al., 2016), and lifespan reduction (Baker et al., 2016; Xu et al., 2018). This evidence strongly suggests that cellular senescence is a key player in the biological aging process. In the past 5 years, a new class of drugs has been developed that can specifically eliminate senescent cells (termed senolytics) (Niedernhofer and Robbins, 2018). These drugs only require intermittent administration (which greatly reduces costs and chances of severe side effects) and have shown promise in collectively delaying a range of age-related diseases (Tchkonia and Kirkland, 2018). Importantly, a number of clinical trials have started to test the safety and efficacy of senolytics in various age-related conditions in humans. Very recently, the first open label pilot study using senolytics showed that intermittent administration of a senolytic cocktail [dasatinib (D) + quercetin (Q)] is relatively safe with minimal severe side effects. In this study, the senolytic cocktail resulted in significant and clinically meaningful improvement in the physical function of all 14 older adults with idiopathic pulmonary fibrosis (IPF) (Justice et al., 2019b). This rapid and exciting progress demonstrates the significant clinical potential of targeting senescent cells—or other hallmarks of aging—in treating age-related diseases, including COVID-19. A recent study has demonstrated that aged mice experience a nearly 100% mortality when exposed to SARS-CoV-2-related mouse β-coronavirus and that the administration of senolytics significantly reduces such mortality by decreasing senescent cell burden (Camell et al., 2021).

Targeting the hallmarks of aging does not necessarily need to be done *via* therapeutics. Lifestyle factors are also associated with the hallmarks of aging. As mentioned earlier, while African Americans have been disproportionately impacted by COVID-19 compared to their white counterparts, but when accounting for sociodemographic factors and chronic diseases these disparities go away (Price-Haywood et al., 2020). This indicates that identifying and addressing the underlying sociodemographic factors may alleviate the severity of the hallmarks of aging that contribute to advanced biological age. Additionally, life changes in older adults—such as diet, lack of exercise, and stress attributed to isolated living—may influence microbiome dysfunction (Stehle et al., 2012; Ghosh et al., 2015; O'toole and Jeffery, 2015). As the network topology of the biological hallmarks of aging is so intertwined, negative lifestyle factors can have detrimental impacts on health, but making lifestyle changes that positively influence one hallmark may potentially be integrated into therapeutic strategies to alleviate biological aging on a broad scale. Therefore, multidisciplinary and systems-based approaches are necessary to account for lifestyle factors when evaluating geroscience-guided approaches to COVID-19.

## Conclusion

There is growing evidence that biological aging represents a powerful and potentially modifiable risk factor for severe COVID-19 resulting in hospitalization and death upon exposure to the SARS-CoV-2 virus. However, our review of the literature indicates that viewing the impact of aging and chronic diseases through the prism of any one single biological mechanism provides a picture that is incomplete and may not move the field forward. All biological hallmarks of aging intricately interlink and are cross-dependent on each other. Moreover, nearly all such hallmarks may potentially contribute to the enhanced vulnerability to severe COVID-19 seen with biological aging. Given the broad spectrum of expertise and the massive amounts of data necessary to understand how these hallmarks of aging interact to drive COVID-19 severity, we propose an approach that is far more multidisciplinary and systems-based emphasizing network topology of biological aging and geroscience-guided approaches to COVID-19. This approach seeks to examine the manner in which biological hallmarks interact with each other in humans and jointly exert their effects across varied tissues and organ systems in contributing to varied disease processes. This type of integrative systems-based approach requires a multidisciplinary approach with the participation of investigators willing and capable of looking beyond their usual “comfort zone.” While such efforts require considerable dedication and time they can yield important benefits. Beyond the context of COVID-19, it will also in our view help prepare us for future pandemics when confronted with future, novel pathogens.

## Data Availability

The original contributions presented in the study are included in the article/Supplementary Material, further inquiries can be directed to the corresponding author.
